# Alpha-type phospholipase A_2_ inhibitors from snake blood

**DOI:** 10.1186/s40409-017-0110-2

**Published:** 2017-03-23

**Authors:** Norival A. Santos-Filho, Claudia T. Santos

**Affiliations:** 1 Institute of Chemistry, São Paulo State University (UNESP – Univ Estadual Paulista), Araraquara, SP Brazil; 2 School of Pharmaceutical Sciences, São Paulo State University (UNESP – Univ Estadual Paulista), Araraquara, SP Brazil

**Keywords:** Phospholipases A_2_, Myotoxin, Myotoxin inhibitor, αPLI, Snake blood

## Abstract

It is of popular and scientific knowledge that toxins from snake venom (among them the PLA_2_ and myotoxins) are neutralized by various compounds, such as antibodies and proteins purified from animal blood. Venomous and nonvenomous snakes have PLA_2_ inhibitory proteins, called PLIs, in their blood serum. One hypothesis that could explain the presence of these PLIs in the serum of venomous snakes would be self-protection against the enzymes of their own venom, which eventually could reach the circulatory system. However, the presence of PLIs in non-venomous snakes suggests that their physiological role might not be restricted to protection against PLA_2_ toxins, but could be extended to other functions, as in the innate immune system and local regulation of PLA_2_s. The present study aimed to review the currently available literature on PLA_2_ and myotoxin alpha inhibitors present in snake plasma, thus helping to improve the research on these molecules. Furthermore, this review includes current information regarding the mechanism of action of these inhibitors in an attempt to better understand their application, and proposes the use of these molecules as new models in snakebite therapy. These molecules may help in the neutralization of different types of phospholipases A_2_ and myotoxins, complementing the conventional serum therapy.

## Background

Between 2009 and 2013, the World Health Organization (WHO) included envenomation by snakes among the neglected tropical diseases given the large number of accidents, the complexity of the clinical condition and the fact that the most affected population consists mainly of workers from poor rural communities in tropical countries [1–4]. However, nowadays experts in Toxinology call on WHO and governments to re-establish snakebite as a neglected tropical disease, since each year, approximately 421,000 cases of snakebite occur, of which approximately 20,000 result in death [5].

Generally, the lethality of bites is low, though the frequency of sequelae related to local complications is higher, especially when associated with risk factors such as the use of a tourniquet, bite in extremities (fingers and toes) and delayed treatment [6]. It is important to note that some sequelae – especially those that lead to partial or total limb amputation – despite been a public health problem, also constitute social problems, since they may provoke various disorders, including the disability to work [5]. Snake venoms are a complex mixture of components, and more than 90% of their dry weight consists of proteins with a large variety of enzymes, and a non-protein portion comprising carbohydrates, lipids, metals, free amino acids, nucleotides and others [7]. The protein components of snake venoms include cytotoxins, cardiotoxins, nerve growth factors, lectins, enzyme inhibitors and various enzymes, such as phospholipase A_2_ (PLA_2_), metalloproteases, serine proteases, phosphodiesterases, cholinesterases, aminotransferases, L-amino acid oxidases, catalases, ATPases, hyaluronidases, etc. [8].

Thus, considering the search for natural inhibitors that neutralize snake venom toxins is of extreme importance for the production of more efficient antivenoms, the present study aims to review the currently available literature on alpha inhibitors present in snake plasma, thus helping to improve the current knowledge about these molecules.

## Phospholipases A_2_ (PLA_2_)

Phospholipases are a superfamily of enzymes that act on phospholipids in the cell membrane leading to their cleavage in fatty acids and lysophospholipids. Phospholipases A_2_ (PLA_2_) (EC 3.1.1.4) were the first phospholipases to be known and their discovery was based on observation of the action of pancreatic fluid of mammals and snake venom in the hydrolysis of phosphatidylcholine [9].

These enzymes play an important role in several cellular functions including maintenance of cellular phospholipids, generation of prostaglandins (PGs) and leukotrienes, cell proliferation and muscle contraction. Furthermore, it is known that these enzymes are involved in human inflammatory processes and due to their central role in many cellular processes, they have been extensively studied [7, 10–12].

The PLA_2_s are a superfamily of enzymes belonging to 16 groups and subgroups that can also be divided into six distinct types: the secreted PLA_2_ (sPLA_2_), among them PLA_2_s found in snake venoms; the cytosolic PLA_2_ (cPLA_2_); the Ca^2+^ independent PLA_2_s (iPLA_2_); the acetyl-hydrolases activating factors of platelets (PAF-AH); lysosomal PLA_2_ and the lipoprotein-associated phospholipase A_2_ (Lp-PLA_2_) [13, 14].

According to Schaloske and Dennis [13] and Dennis et al. [14], the sPLA_2_s are enzymes with a molecular weight between 14,000 and 18,000 Da, usually containing from 5 to 8 disulfide bridges. These enzymes have a histidine in their active site and require the presence of Ca^2+^ ion for catalysis. The phospholipase A_2_ from groups IA, IB, IIA, IIB, IIC, IID, IIE, IIF, III, V, IX, X, XIA, XIB, XII, XIII and XIV are representatives of sPLA_2_s.

The PLA_2_s from snake venoms (svPLA_2_s) are classified into groups I and II, and those from the Viperidae family belong to group IIA [11, 13–15]. The svPLA_2_s belonging to group IIA are subdivided into subgroups based on the presence of a conserved residue on position 49, being the most studied: (i) PLA_2_s Asp49, enzymes that usually have high catalytic activity, and (ii) homologous PLA_2_s (or PLA_2_-like) Lys49, which have no enzymatic activity [16, 17]. It is important to point out that other variants in snake venom group II PLA_2_s have been reported, e.g., Ser49, Asn49 and Arg49 [18–23].

Interestingly, despite having no catalytic activity, the homologous PLA_2_s Lys49 have a wide variety of pharmacological and/or toxic effects, including myotoxicity, cytotoxicity, antibacterial, antifungal, muscle necrotic and anticoagulant activities [7, 24–27]. According to some authors, the main structural domain responsible for the toxic effect, particularly cytotoxic, in homologous Lys49-PLA_2_ is the C-terminal region (amino acids 115–129) [27].

## PLA_2_ inhibitory proteins (PLIs) from snake blood

Venomous and non-venomous snakes have PLA_2_ inhibitory proteins, called PLIs, in their blood serum [28–30]. These PLA_2_ inhibitory proteins are produced by the liver, as indicated by Northern blot analysis and RT-PCR analysis of genetic material extracted from different tissues. This PLI production by the liver (and not by the venom glands or other organ) makes it possible for these proteins to enter the bloodstream, since the liver is the main organ producing plasma proteins, thus improving and accelerating the protection mechanism against poisoning [31–33]. Furthermore, it has been known that some secreted PLA_2_ receptors, which have structural similarity with PLIs, also exist in soluble forms, showing that PLIs, as well as PLA_2_ endogenous receptors, could have a regulatory role of proinflammatory activity of sPLA_2_s [34].

Several PLIs were purified from the plasma of different species of snakes, and their structures have been determined [28–30, 34, 35]. So far, for the isolation of PLA_2_ inhibitors described in the literature, two different methods were used. One of these purification methods is the bioaffinity chromatography, which is based on the immobilization of different proteins, PLA_2_ in this case (for example BthTX-I and BthTX-II, from *Bothrops jararacussu*), on a stationary phase [32, 36–40]. Another method used in purification of PLIs from snake plasma is a sequence of chromatographic steps such as gel filtration, ion exchange and hydrophobic chromatography [35, 41, 42].

The blood used for plasma separation is typically collected by cardiac puncture, by puncturing the tail vein or after decapitation of the snake. It is noteworthy that in recent years concern about the ethics in the use animals for experimentation is growing and therefore the least aggressive method that does not require animal death is the blood collection from the tail vein of the snake, being the most indicated. After collecting the blood, plasma and serum are separated, then plasma is lyophilized and stored. During purification, the inhibitory activity of these PLIs is monitored by biological assays based on inhibition activity of PLA_2_ and myotoxins, depending on the inhibitor of interest.

The PLA_2_ and myotoxin inhibitors from the blood of snakes are globular, acid and oligomeric proteins, which form soluble complexes with PLA_2_ and myotoxins, thus inhibiting the action of these molecules [34, 43–46]. Blood inhibitors found in snakes are classified into types alpha (α), beta (β) and gamma (γ) according to structural aspects [30, 47, 48].

One of the PLIs classes, the βPLIs, have repeated leucine-rich structures and show similarity to human α2-glycoprotein [49]. βPLIs inhibit only basic group II PLA_2_s isolated from snake venoms and have been isolated from plasma of *Agkistrodon blomhoffii siniticus*, *Elaphe quadrivirgata* and *E. climacophora* snakes, which belong to the Viperidae and Colubridae family [33, 49, 50].

Another type of PLIs, known as γPLIs, is the most abundant to date. The γPLIs are acidic glycoproteins with a mass of 90–130 kDa consisting of 3 to 6 noncovalent subunits. Their amino acid sequences contain two sets of standards cysteine residues, responsible for the formation of the three-finger motif [51]. This type of inhibitor has been reported in different snakes, as *Crotalus durissus terrificus* [52–54], *Naja naja kaouthia* [55, 56], *Agkistrodon blomhoffii siniticus* [57], *Trimeresurus flavoviridis* [58], *Laticauda semifasciata* [59], *Elaphe quadrivirgata* [60]*, E. climacophora* [50], *Cerrophidion godmani* [32], *Notechis ater*, *Notechis ater serventyi* [61], *Oxyuranus scutellatus* and *O. microlepidotus* [61], *Pseudonaja textilis* [61], *Python reticulates* [62], *Notechis scutatus* [63], *Lachesis muta muta* [64], *Protobothrops flavoviridis* [65], *Bothrops alternatus, B. erythromelas, B. jararaca, B. moojeni, B. neuwiedi* [51], *Bothrops jararacussu* [39] and *Crotalus durissus collilineatus* [66] and these γPLIs appear to be less specific, since they inhibit PLA_2_ from groups I, II and III.

## Alpha-type PLA_2_ inhibitor

The alpha-type PLA_2_ inhibitors (αPLIs) from the snake blood are found mainly as trimers in solution and have a region with high similarity with the carbohydrate recognition domain (CRD) of C-type lectins and pulmonary surfactant protein [30, 36, 37, 40, 67–70]. This region covers approximately 67% of the primary sequence of the monomers of αPLIs and is the most conserved portion of these molecules, with approximately 46% of sequence identity between species [30]. The CRD of αPLIs lacks the amino acid residues involved in Ca^2+^ binding, making the interaction with their respective ligands Ca^2+^-independent [40, 42]. Moreover, several studies have shown that the carbohydrate motif present in αPLIs is not necessary for the connection with PLA_2_ [32, 38].

## αPLIs studied to date

Various αPLIs were purified to date (Table 1), such as the plasma PLI from the snake *Trimeresurus flavoviridis*, which was purified by a combination of chromatographic steps through Sephadex gel filtration column G-200, DEAE-cellulose anion exchange and Blue Sepharose CL-6B [41]. The purified inhibitor was found as a glycoprotein with an approximately molecular weight of 100,000 Da, with non-homologous subunits of approximately 20,000 to 24,000 Da. Subsequently, it was verified the ability of this inhibitor to interact with venom phospholipase A_2_ of *T. flavoviridis*, and *Agkistrodon halys blomhoffii*, besides the enzyme and the porcine pancreatic phospholipase C of *Bacillus cereus*. According to Kogaki et al. [41], this inhibitor showed specificity to *T. flavoviridis* PLA_2_, and an independent inhibitory activity of Ca^2+^.

**Table 1:** Alpha-type PLA_2_ inhibitors (αPLIs) studied to date

Purification method	Source	Name	Reference
Sequential chromatography on Sephadex G-200, DEAE-cellulose and Blue Sepharose CL-6B	*Trimeresurus flavoviridis*	*Tft*PLIα	[41]
Sequential chromatography on Sephadex G-200, Mono Q and Blue Sepharose CL-6B	*Agkistrodon blomhoffii siniticus*	*Gb*PLIα	[42]
Affinity chromatography with Sepharose-immobilized myotoxins (myotoxins I, II, III and IV from *B. asper* venom)	*Bothrops asper*	BaMIP	[73]
Affinity chromatography containing myotoxin II isolated from *C. godmani* venom, coupled to CNBr-activated Sepharose 4B	*Cerrophidion godmani*	CgMIP-II	[32]
Affinity chromatography containing *B. moojeni* MjTX-II coupled to CNBr-activated Sepharose 4B	*Bothrops moojeni*	BmjMIP	[36]
Sequential chromatography on Hi-trap Blue, Mono Q, and Superdex 200	*Elaphe quadrivirgata*	*Eq*PLIα	[68]
Affinity chromatograph containing myotoxins I and II from *A. nummifer* coupled to NHS-activated column	*Atropoides nummifer*	AnMIP	[37]
Affinity chromatography containing *B. jararacussu* BthTX-I coupled to CNBr-activated Sepharose 4B	*Bothrops jararacussu*	αBjussuMIP	[38]
Sequential chromatography Blue Sepharose 6FF, Q-Sepharose and Superdex 200 HR10/30	*Elaphe climacophora*	PLIα	[50]
Affinity chromatography containing BthTX-I, from *B. jararacussu*, coupled to CNBr-activated Sepharose 4B	*Bothrops alternatus*	αBaltMIP	[40]

Afterward, Inoue et al. [67] purified two distinct but homologous subunits (PLIα-A and PLIα-B) of the PLI from *Trimeresurus flavoviridis*. These subunits were separated by reversed-phase HPLC and showed molecular weights around 21,000–22,000 Da when glycosylated and 17,000 after deglycosylation. Furthermore, the sequences were significantly homologous to CRD portions of pulmonary surfactant apoprotein and animal lectins. Then, Shimada et al. [71] studied this αPLI, which was purified into different subspecies of two homologous subunits. Before this work, it was expected that this αPLI was a tetramer, composed of two molecules of αPLI-A and two molecules of αPLI-B [67]. However, in this last study, it was showed that this αPLI is a trimeric protein. Curiously, all the αPLIs except that from *P. flavoviridis* are multimers composed of a single subunit.

Ohkura et al. [42] purified an alpha inhibitor from the snake *Agkistrodon blomhoffii siniticus*, using a similar method described by Kogaki et al. [41]. In this case, this αPLI purification was performed by sequential chromatography through Sephadex G-200 column, Mono Q and Sepharose Blue CL-6B. The purified PLI showed up as a glycoprotein with a molecular mass from 75,000 to 24,000 Da for the trimer and the monomer.

After, Inoue et al. [72] studied the specificity of the two previously purified (and cited above) PLA_2_ inhibitors from *T. flavoviridis* and *A. b. siniticus* plasma, purified by Kogaki et al. [41], and Ohkura et al. [42], respectively. Both αPLI showed a high specificity for group II acidic PLA_2_s from their own venom. In this work, the authors draw a parallel between PLI from snake plasma and PLA_2_ receptors of rabbit, bovine, and human, suggesting that the CRD-like domain would be involved in the binding to the PLA_2_ molecule.

Regarding the αPLI from *Bothrops genus*, other α inhibitors were purified, for example, BaMIP, a PLI isolated from the plasma of *Bothrops asper* by affinity chromatography in Sepharose 4B CNBr-activated with myotoxins immobilized [73]. BaMIP presented monomers with a molecular weight of approximately 24,000 Da and a structure in solution composed of five subunits. The BaMIP showed inhibition on myotoxic, edema and cytolytic activity of the myotoxins I and III of *B. asper* snake. Structural studies have also shown that BaMIP, as well as all α phospholipase A_2_ inhibitors has a homologous domain to CRD of C-type lectins.

Another snake inhibitor studied is CgMIP-II, an αPLI, purified from plasma of *Cerrophidion (Bothrops) godmani* snake by affinity column containing myotoxins [32]. The inhibitor is an acidic protein (pI 4.0), glycosylated, the monomeric subunits with a molecular weight between 20,000 Da and 25,000 Da, forming a polymer of about 180,000 Da.

Soares et al. [36] purified a protein that neutralizes the enzymatic, toxic and pharmacological activity of a variety of toxins (acidic or basic) of different venoms. This inhibitor, called BmjMIP, was isolated from the plasma of the snake *Bothrops Moojeni*, by affinity chromatography. BmjMIP presented similar biochemical and structural characteristics to those already described for αPLIs, besides being stable at a wide range of pH and temperature.

Okumura et al. [68] purified the αPLI-like protein (PLIα-LP) from a non-venomous snake *E. quadrivirgata* serum by sequential chromatography on Hi-trap Blue, Mono Q and Superdex 200 columns. The PLIα-LP showed the highly conserved C-type lectin-like domain (CTLD) and 51 kDa, being a trimer. Although this protein has about 70% similarity with other inhibitors previously studied, this protein did not demonstrate any inhibitory activity against different PLA_2_s. It is important to cite that Shirai et al. [50] also purified an αPLI-like protein (PLIα-LP) from *E. climacophora* snake. According to Okumura et al. [68], the high homology with αPLIs and the lack of inhibitory activity on αPLI-like proteins may provide important information concerning the structure/function of these αPLIs.

Quirós et al. [37], purified an αPLI (AnMIP) from the plasma of *Atropoides nummifer* by affinity matrix, prepared by coupling a mixture of myotoxins I and II from *A. nummifer* to an NHS-activated column. According to the work, this trimeric inhibitor neutralized the activity of basic PLA_2_ myotoxins and showed specificity towards group II PLA_2_, either belonging to the catalytically active (Asp49 PLA_2_) or inactive (Lys49 PLA_2_-like) subtypes.

Oliveira et al. [38] and Santos-Filho et al. [40] purified two different αPLIs (named αBjussuMIP and αBaltMIP), from *B. jararacussu* and *B. alternatus*, respectively. These molecules were purified through affinity chromatography using BthTX-I immobilized on Sepharose gel and neutralize enzymatic, toxic and pharmacological activities of several phospholipases A_2_. Santos-Filho et al. [74, 75] subsequently expressed an active recombinant alpha inhibitor, named rBaltMIP, in *Pichia pastoris* heterologous system. According to these works, heterologous expression would enable large-scale obtainment of these αPLI, thus allowing further investigations for the elucidation of possible mechanisms of inhibition of PLA_2_s, which have not yet been fully clarified.

## Mechanism of action of αPLIs

In the last 30 years, several studies have been published aiming to biochemically, structurally and functionally characterize αPLIs. However, the mechanism of action of these αPLIs is still unknown. Some authors have suggested that the αPLI/PLA_2_ binding site is probably related to the CRD region of the molecule, which recognizes and binds to the enzyme, preventing its toxic activity. One factor that supports this idea is that these CRD domains are present in endogenous PLA_2_ receptors, such as the human receptor of group I pancreatic PLA_2_ and receptors of group II secretory PLA_2_ from rabbits, mice, cattle and humans [38, 73, 76–78]. Nevertheless, the molecular nature of the interaction between the CRD region and PLA_2_ is still unknown and efforts towards the elucidation of the structure of αPLIs and their complexes are being performed [30].

Studying the deletion of amino acid residues, Nobuhisa et al. [79] mapped the interaction between an αPLI and an acidic PLA_2_ from *T. flavoviridis*, noting that the binding capacity was more restricted to the C-terminal region between residues 136 and 147. In this region, two hydrophobic tripeptides and Tyr144 residue appear to be involved in the interaction PLI/PLA_2_ [37, 69, 79].

Thereafter, Okumura et al. [69] studied the relationship of the structure/function of the αPLI previously purified from the snake *Agkistrodon blomhoffii siniticus*, named GbPLIα, and the αPLI-like protein EqPLIα-LP, purified from the nonvenomous snake *Elaphe quadrivirgata,* and which does not show inhibitory activity against PLA_2_s [42, 68]. In that work, by constructing chimeric proteins, they mapped important residues to the inhibitory activity of the αPLIs; for example, the region 13-36 of the neck C-terminal portion of the trimer. Interestingly, the region found as the responsible for PLA_2_ inhibition was distinct from the carbohydrate-binding site. Furthermore, other residues were pointed as candidate, including Asn26, Lys28, Asp29, and Tyr144 [69].

According to Okumura et al. [69], the trimer is formed through the interactions of the helical neck regions, forming a central pore, responsible for PLA_2_ binding. Furthermore, as Tyr144 is expected to be located in this central pore, this residue may be one of the responsibles for the direct interaction to the PLA_2_ molecule. In a complementary study, Nishida et al. [70] created heterotrimers of αPLI composed of two different subunits derived from the recombinant GbPLIα, EqPLIα-LP, and chimeras of GbPLIα-EqPLIα-LP homotrimers, in order to estimate the contribution of each subunit to the total inhibitory activity as a trimeric PLA_2_ inhibitory protein. Summing up, in this work, it was observed, once more, the importance of the residues 13–36 for the trimer formation, and consequently for the αPLI inhibitory activity. Furthermore, the interactions between residues Glu23 and Lys28 of GbPLIα were also suggested to be important to stabilize the trimeric structure.

**Fig. 1:**
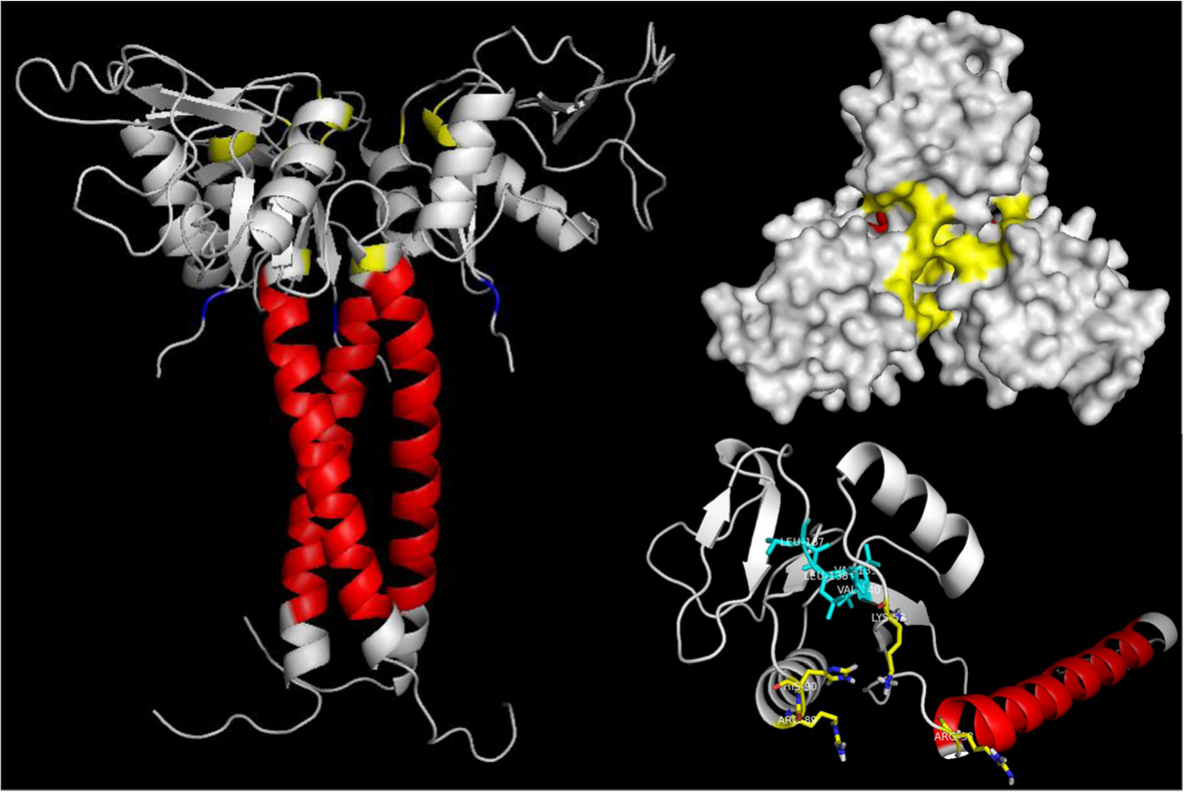
*In silico* model of αBaltMIP trimer (available at Model Archive database under the DOI 10.5452/ma-a4btt) and αBaltMIP monomer (available at Model Archive database under DOI 10.5452/ma-a2iil) with a detailed view of the central pore (*yellow*), highlighting the four conserved cationic residues R38, K52, R89 and H90. In addition, the hydrophobic core (*cyan*), the 13–36 residues of the neck C-terminal region (*red*) and the Y144 (*blue*) are depicted

Lastly, in a recent study, Estevão-Costa et al. [80] studied the importance of αPLI trimerization for the binding and inhibition to acidic PLA_2_s. Furthermore, they suggested that the central pore, which is composed by positive charged residues, especially Arg57, Lys71, Arg108 and His109, could be a significant part of the binding site of αPLIs to acidic PLA_2_s. In addition, these authors pointed the importance of the hydrophobic core (Leu158 to Val161), which may be the responsible for the central pore structural integrity. However, the positive surface of the basic PLA_2_ could prevent the PLA_2_/PLI interaction at the central pore and according to these authors, the mechanism of inhibition of basic PLA_2_ by αPLIs remains to be understood. It is interesting to point out that, considering the sequence of the native protein, obtained through Edman degradation sequencing [40], the numbering of central pore important residues should be Arg38, Lys52, Arg89 and His90 (Fig. 1).

So far, it is possible to observe that the mechanism of action of these inhibitors and the region responsible for their inhibitory properties are not yet fully elucidated in the literature, requiring further study concerning these macromolecules and their interactions with PLA_2_s.

## Potential complement of antiophidic serum therapy

Currently, antiserum composed of specific immunoglobulins is the only treatment for snake envenomation, but there are ongoing issues with availability, effectiveness and dosing [81–83]. These antivenoms neutralize the toxicity and lethality of specific venoms, but their administration is often related with significant clinical side effects [84, 85]. Additionally, the production of antivenoms is associated with high costs related to animal maintenance and also comes across animal welfare concerns, which instigates the search for innovative products for snakebite therapy [82, 86].

Interestingly, the production of specific antivenom was started by Vital Brazil in the 1900’s and it was Vital Brazil who also discovered the effectiveness of the polyvalent antivenom [87, 88]. At that time, antivenom was prepared with crude plasma of hyperimmunized animals. However, it was thereafter discovered that antibodies (immunoglobulins) were the active therapeutic molecules responsible for the action of the antivenom. Therefore, only the antibodies started to be purified and used in antivenom therapy.

Nowadays, despite advances in the production of antivenoms, this production is still similar to the methods originally described by Vital Brazil [87, 88]. Currently, immunoglobulins or immunoglobulin fragments [F(ab’)2 or Fab] purified from serum are used in antivenom [2]. Other innovations have been proposed on traditional antiserum, as the use of the single chain variable fragment (scFv) or the use of recombinant antigen binding domains derived from camelid heavy chain antibodies (VHH) [82, 89–91]. However, there are numerous challenges on antivenom improvement, for example, the high cost of monoclonal antibodies production or the lower affinity and the short serum half-life profiles of some immunoglobulin fragments [82, 92].

Although serum therapy effectively reverses the systemic effects of venom into the victim’s body, avoiding death many times, it has some disadvantages including a number of side effects (anaphylactic shock, renal failure and serum sickness, for example). The inefficiency to combat the local effects of the envenomation (increasing the chances of sequelae in the stricken member), the need for careful storage and the short shelf life of the serum are also other limiting factors.

PLA_2_ enzymes and PLA_2_-like myotoxins are the main responsible for myonecrosis, an important medical complication of snake envenomation, and which, in severe cases can lead to drastic consequences such as permanent loss of tissue or limb amputation. These outcomes provoke severe problems for both the affected individual and public health, since the victim may become incapable of working and lose life quality. In addition, these sequelae burden the public health once they increase the length of hospitalization and surgeries and, in some cases, can lead to early retirement of the individual affected by the envenomation.

The search for natural inhibitors that neutralize snake venom toxins is of extreme importance for the production of more efficient antivenoms, especially considering that several toxins induce weak immunogenic responses, making traditional serum therapy unable to inhibit local effects such as the myotoxicity induced by phospholipases A_2_ and PLA_2_-like enzymes [46, 93].

## Conclusions

In conclusion, the traditional antivenom is not completely able to inhibit local effects of envenomation, mainly caused by myotoxins. Thus, the search for proteins, such as αPLIs, that neutralize myotoxins present in snake venom is extremely important for the production of a more efficient treatment.

## Abbreviations

cPLA2: Cytosolic PLA2
CRD: Carbohydrate recognition domain
CTLD: C-type lectin-like domain
iPLA2: Ca2+ independent PLA2s
Lp-PLA2: Lipoprotein-associated phospholipase A2
PAF-AH: Acetyl-hydrolases activating factors of platelets
PG: Prostaglandin
PLA2: Phospholipase A2
PLI: PLA2 inhibitory proteins
sPLA2: Secreted PLA2
αPLI: Alpha-type PLA2 inhibitor
